# The community structure of *Methanomassiliicoccales* in the rumen of Chinese goats and its response to a high-grain diet

**DOI:** 10.1186/s40104-017-0178-0

**Published:** 2017-06-01

**Authors:** Wei Jin, Yanfen Cheng, Weiyun Zhu

**Affiliations:** Jiangsu Province Key Laboratory of Gastrointestinal Nutrition and Animal Health; Laboratory of Gastrointestinal Microbiology, College of Animal Science and Technology, Nanjing Agricultural University, Nanjing, 210095 China

**Keywords:** Community structure, Diet, Goat, *Methanomassiliicoccales*, Pyrosequencing

## Abstract

**Background:**

The newly proposed methanogenic order ‘*Methanomassiliicoccales*’ is the second largest archaeal population in the rumen, second only to the *Methanobrevibacter* population. However, information is limited regarding the community of this new order in the rumen.

**Methods:**

This study used real-time PCR and 454 pyrosequencing to explore the abundance and community composition of *Methanomassiliicoccales* in the rumen of Chinese goats fed a hay (0% grain, *n* = 5) or a high grain (65% grain, *n* = 5) diet.

**Results:**

Real-time PCR analysis showed that the relative abundance of *Methanomassiliicoccales* (% of total archaea) in the goat rumen was significantly lower in the high-grain-diet group (0.5% ± 0.2%) than that in the hay-diet group (8.2% ± 1.1%, *P* < 0.05). The pyrosequencing results showed that a total of 208 operational taxonomic units (OTUs) were formed from ten samples at 99% sequence identity. All the sequences were identified as Methanomassiliicoccaceae at the family level, and most of the sequences (96.82% ± 1.64%) were further classified as Group 8, 9, and 10 at the *Methanomassiliicoccales* genus level in each sample based on the RIM-DB database. No significant differences were observed in the number of OTUs or Chao1’s, Shannon’s or Pielou’s evenness indexes between the hay- and high-grain-diet groups (*P* ≥ 0.05). PCoA analysis showed that diet altered the community of *Methanomassiliicoccales*. At the genus level, the relative abundances of Group 10 (67.25 ± 12.76 vs. 38.13 ± 15.66, *P* = 0.012) and Group 4 (2.07 ± 1.30 vs. 0.27 ± 0.30, *P* = 0.035) were significantly higher in the high-grain-diet group, while the relative abundance of Group 9 was significantly higher in the hay-diet group (18.82 ± 6.20 vs. 47.14 ± 17.72, *P* = 0.020). At the species level, the relative abundance of Group 10 sp. (67.25 ± 12.76 vs. 38.13 ± 15.66, *P* = 0.012) and Group 4 sp. MpT1 (2.07 ± 1.30 vs. 0.27 ± 0.30, *P* = 0.035) were significantly higher in the high-grain-diet group, while the relative abundance of Group 9 sp. ISO4-G1 was significantly higher in the hay-diet group (12.83 ± 3.87 vs. 42.44 ± 18.47, *P* = 0.022).

**Conclusions:**

Only a few highly abundant phylogenetic groups dominated within the *Methanomassiliicoccales* community in the rumens of Chinese goats, and these were easily depressed by high-grain-diet feeding. The relatively low abundance suggests a small contribution on the part of *Methanomassiliicoccales* to the rumen methanogenesis of Chinese goats.

## Background

Recently, a large group of archaea closely related to the *Thermoplasmatales* has been proposed as a new methanogenic order ‘*Methanomassiliicoccales*’ [1, 2]. Two synonymous names, ‘Rumen Cluster C’ (RCC) and ‘*Methanoplasmatales*’, were previously used for this new order before the formal name was proposed [2–4].

St-Pierre and Wright [5] reviewed the representation of gastrointestinal methanogens in a variety of host species based on 16S r RNA gene clone library analysis and found wide variations in the abundance of *Methanomassiliicoccales* in the rumen. In most cases, *Methanomassiliicoccales* accounted for less than 5% of the archaea in the rumens of sheep, water buffalo, yaks, dairy cattle, and other ruminant species [5]. In several cases, including reindeer (Svalbard), beef cattle (Hereford-cross, potato diet), and sheep (Merino, Queensland, Australia), *Methanomassiliicoccales* accounted for more than 50% of archaea in the rumen [5]. Recently, Seedorf et al. [6] used 454 pyrosequencing to study the methanogenic communities in the rumens of New Zealand sheep and cattle fed with pasture (ryegrass and white clover). They reported a mean relative abundance of *Methanomassiliicoccales* of about 10.4%, but this value exceeded 40% in some treatment groups [6]. This high degree of abundance suggests the significant involvement of the *Methanomassiliicocales* in rumen methanogenesis, but their exact role remains to be established. Interestingly, increasing evidence shows that the *Methanomassiliicoccales* do not possess the genes required for the first six steps of hydrogenotrophic methanogenesis [7]. Instead, methane is produced by via the hydrogen-mediated reduction of methyl-group-containing compounds (e.g., methylamines and methanol) [8–10].

*Methanomassiliicoccales*-affiliated 16S rRNA genes have been found in a wide range of anoxic environments [11], including marine habitats, rice paddy fields, anaerobic digestors, and the intestinal tracts of termites, mammals, and humans. Most recently, Söllinger et al. [10] confirmed a clade-specific habitat preference on the part of *Methanomassiliicoccales* and divided this order into two clades, the ‘gastrointestinal tract’ (GIT) clade and the ‘environmental’ clade, based on phylogenetic and genomic analyses of methanogenic sequences from wetlands and animal intestinal tracts. The authors also reported larger genomes and a larger number of genes encoding anti-oxidative enzymes in the ‘environmental’ clade than in the ‘GIT’ clade representatives.

The taxonomy of *Methanomassiliicoccales* remains incomplete due to the lack of type strains, which has limited the study of the community of this new archaeal order [12]. Seedorf et al. [12] constructed a 16S rRNA gene database (RIM-DB) for the phylogenetic analysis of archaea from the gastrointestinal tract. This database clustered the sequences of *Methanomassiliicoccales* into twelve groups at the genus level, thereby enabling the analysis of the community structure of this order. Using this database, Seedorf et al. [6] reported that the members of *Methanomassiliicoccales* in New Zealand sheep and cattle were mainly located in Groups 10, 11, and 12.

China has more than 140 million goats (China Statistical Yearbook 2015), which is an important contributor to the production of the greenhouse gas methane. For the development of methane-mitigation tools, it is important to understand the methanogen community and identify the dominant methanogens in the rumen of Chinese goats [6]. St-Pierre and Wright reviewed the diversity of gut methanogens in herbivorous animals and concluded that the population structure of methanogens was strongly influenced by diet and host species [5]. Thus, we hypothesized that the community of the new established methanogenic order ‘*Methanomassiliicoccales*’ in the rumens of Chinese goats was distinct from that in the other ruminants examined in the previous studies and also affected by the two main dietary types in China. In the present study, we used real-time PCR and 454 pyrosequencing to determine the abundance and the composition of *Methanomassiliicoccales* in the rumens of Chinese goats based on the RIM-DB database. Our findings differ from those previously reported for New Zealand sheep and cattle because the *Methanomassiliicoccales* community in the rumens of Chinese goats consists mainly of members of Groups 8, 9, and 10.

## Methods

### Animal, diet and experimental design

This animal experiment was carried out at the experimental station of Nanjing Agricultural University in Jiangsu Province, China. The experimental design was previously described in detail [13]. Briefly, ten male goats with rumen fistula (Boer × Yangtze River Delta White; 2–3 years old) were used and placed in individual pens (1.2 m× 1.2 m) with free access to water. These animals were randomly assigned to one of two groups and fed either a hay diet (0% grain, *n* = 5) or high-grain diet (65% grain, *n* = 5). The body weights of the goats were 29.8 ± 0.9 kg for the hay-diet group and 30.0 ± 1.1 kg for the high-grain-diet group at the beginning of the trial. The diets (750 g dry matter per animal per day) were offered in equal amounts at 08:30 and 16:30 daily during the trial (7 wks). The nutrient compositions of the diets are presented in Table 1. The metabolic energy intake was slightly above that required for the maintenance of goats in the hay group and permitted a growth rate of 200 g/d in the high-grain group (30 kg body wt).

**Table 1:** Ingredient and nutrient composition of the diets

Item	Hay diet	High grain diet
Ingredient composition, % DM		
Chinese wildrye	81.00	30.00
Alfalfa	15.00	0
Corn meal	0	45.00
Wheat meal	0	20.00
Soybean	0	1.10
CaCO_3_	0.50	0.95
NaCl, Salt	0.80	0.65
CaHPO_4_	1.70	1.20
Mineral and vitamin supplement	1.00	1.00
NaHCO_3_	0	0.10
Nutrient composition		
Metabolic energy, MJ/kg DM	8.31	11.31
Crude protein, % DM	10.06	10.06
Crude fat, % DM	3.55	3.59
Crude fiber, % DM	30.17	11.18
Neutral detergent fiber, % DM	57.01	25.23
Acid detergent fiber, % DM	35.72	13.55
Crude ash, % DM	10.62	6.52
Starch, % DM	ND^a^	58.23

^a^Not determined, but considered equal to 0

### Sample collection

On d 50, the goats were slaughtered at a local slaughterhouse 4–5 h after the morning feeding. Immediately after slaughter, a representative sample (solid and liquid) from five sections of whole-rumen digesta was collected and homogenised in a soybean milk blender. Then samples were frozen and stored at −80 °C.

### DNA extraction

DNA was extracted from a representative 1 mL of rumen content using a FastPrep®-24 Instrument (MP Biomedicals, South Florida, USA) and processed (bead beat) at a setting of 5 for 2 min, combined with cetyltrimethylammonium bromide and phenol-chloroform-isopentanol extraction [14]. The DNA in the solution was precipitated with ethanol. Then, the pellets were suspended in 50 μL Tris-EDTA buffer. The sample DNA concentrations were quantified using a NanoDrop ND-1000 Spectrophotometer (Nyxor Biotech, Paris, France) and adjusted to yield similar DNA concentrations. The total DNA sample was divided into two portions for 454 pyrosequencing and real-time PCR.

### Real-time PCR

Real-time PCR was performed to quantify the archaea and *Methanomassiliicoccales* according to the method described by Jeyanathan et al. [15] using an Applied Biosystems 7300 Real-Time PCR system (Applied Biosystems, California, USA). The primers for total archaea were 915f 5’-AAG AAT TGG CGG GGG AGC AC, 1386r 5’-GCG GTG TGT GCA AGG AGC. The primers for *Methanomassiliicoccales* were 762f 5’- GAC GAA GCC CTG GGT C, 1099r 5’- GAG GGT CTC GTT CGT TAT. The reaction mixture (20 μL) consisted of 10 μL of SYBR® Premix Ex Tag TM (TaKaRa, Dalian, China), 0.2 μmol/L of each primer, and 2 μL of the template DNA. Real-time PCR amplification for archaea was initiated at 95 °C for 30 s, followed by 40 cycles at 95 °C for 5 s, 59 °C for 30 s, and 72 °C for 30 s. Real-time PCR amplification for *Methanomassiliicoccales* was initiated at 95 °C for 30 s, followed by 40 cycles at 95 °C for 5 s, 56 °C for 30 s, and 72 °C for 30 s. Melting curve analysis was also conducted over a range of 60–95 °C to assess the specificity of the amplification products.

The genomic DNA of the rumen contents was amplified with the two pairs of primers for archaea and *Methanomassiliicoccales*. The reaction mixture (50 μL) consisted of 0.25 μL of TaKaRa Ex Taq® (TaKaRa, Dalian, China), 5 μL 10 × Ex Taq Buffer (Mg^2+^ Plus), 4 μL dNTP Mixture, 0.2 μmol/L of each primer, and 1 μL (10 ~ 20 ng) of the template DNA. PCR amplification for archaea (for *Methanomassiliicoccales*) was initiated at 95 °C for 5 min, followed by 35 cycles at 95 °C for 30 s, 59 °C (56 °C) for 30s, and 72 °C for 30 s, as well as a final extension at 72 °C for 7 min. The amplicons were cloned into *Escherichia coli* JM109 using the pGEM-T Easy vector (Promega). The plasmid containing the amplicon was used to construct a standard for the estimation of the 16S rRNA gene copy number for the total archaea or *Methanomassiliicoccales*. The concentration of the plasmids was quantified using a Qubit dsDNA HS Assay Kit (Invitrogen, Eugene, Oregon, USA) on a Qubit 2.0 Fluorometer (Invitrogen, Carlsbad, CA, USA). The plasmid DNA standard was prepared according to Koike et al. [16]. The copy number of each standard plasmid was calculated based on the DNA concentration and molecular weight of the cloned plasmid.

A 10-fold dilution series of the standard plasmid for the related target was also run with the samples; all samples and standards were run in triplicate. The total numbers of *Methanomassiliicoccales* and archaea per mL were determined using ABI SDS software (Applied Biosystems, Foster City, CA, USA), based on the standard curve, the dilution factor, and the volumes of the DNA extracts.

### Roche 454 pyrosequencing for 16S rRNA gene amplicon of *Methanomassiliicoccales*

The *Methanomassiliicoccales* were low in abundance in the goat rumen samples. Therefore, nested PCR was used to amplify the 16S rRNA gene to obtain a sufficient concentration of PCR product for 454 pyrosequencing. For total methanogens, the 86f/1340r primer pair was used as the outer primers [17]. The PCR reactions were performed in a 20 μL mixture containing 4 μL of 5 × FastPfu Buffer, 2 μL of 2.5 mmol/L dNTPs, 0.8 μL of each primer (5 μmol/L), 0.4 μL of FastPfu Polymerase (TransGen Biotech, Beijing, China), and 10 ng of template DNA. The cycling parameters were as follows: 95 °C for 2 min, followed by 20 cycles at 95 °C for 30 s, 55 °C for 30 s, and 72 °C for 30 s, as well as a final extension at 72 °C for 5 min. The primers for *Methanomassiliicoccales* (762f/1099r), described above, were used as the inner primers. The PCR reactions were performed in a 20 μL mixture containing 4 μL of 5 × FastPfu Buffer, 2 μL of 2.5 mmol/L dNTPs, 0.8 μL of each primer (5 μmol/L), 0.4 μL of FastPfu Polymerase (TransGen Biotech, Beijing, China), and 2 μL of the first round PCR products (diluted 20-fold before use). The cycling parameters were as follows: 95 °C for 2 min, followed by 28 cycles at 95 °C for 30 s, 55 °C for 30 s, and 72 °C for 30 s, as well as a final extension at 72 °C for 5 min. Negative controls were used for both PCR runs (outer primer run and inner primer run) to avoid contamination. After purification using the AxyPrep DNA Gel Extraction Kit (Axygen Biosciences, Union City, CA, U.S.) and quantification using QuantiFluor™ -ST (Promega, U.S.), a mixture of amplicons was used for pyrosequencing on a Roche 454 GS FLX+ Titanium platform (Roche 454 Life Sciences, Branford, CT, U.S.) according to standard protocols [18] at the Majorbio Bio-Pharm Technology Co., Ltd., Shanghai, China.

The raw data were submitted to the Sequence Read Archive, under accession number SRP053014.

### Amplicon sequence data analysis

The analysis was performed following the procedure described by Seedorf et al. [6]. Briefly, data were processed in QIIME [19]. The reads were quality filtered and assigned to the corresponding samples via barcodes, using the *split library.py* script with the “-r -z truncate_remove –s 20” options. Acacia with the default settings was used to correct the error of the sequences. Chimeras were checked using the *parallel_identify_chimeric_seqs.py* script with the parameters “– d 4 and –n 2” and RIM-DB as a reference database [12] and removed using the *filter_fasta.py* script. Sequences were clustered into operational taxonomic units (OTUs) by the *pick_otus.py* script with the default clustering method UCLUST at a distance of 0.01. Representative sequences of OTUs were obtained and classified using the *parallel_assign_taxonomy_blast.py* script with RIM-DB as a reference database for taxonomic assignment. OTU tables were generated using the *make_otu_table.py* script. OTUs that contained less than ten sequences and were not classified as *Methanomassiliicoccales* were removed from the OTU tables. The Chao1 richness index was calculated according to Chao and Bunge [20]. Shannon diversity indices were calculated according to Shannon [21]. Pielou’s evenness index was calculated according to Pielou [22]. The principal coordinates analysis (PcoA) was performed via the unweighted UniFrac distance method [23]. Before the following analysis was performed, the OTU tables were filtered. OTUs that occurred in fewer than three samples were removed from the OTU tables. The predominant OTUs (≥5% of the relative abundance in one of the 10 samples) were used to form the heat map. Hierarchical clustering based on the predominant OTUs was performed with HemI 1.0 software as described by Deng et al. [24].

### Phylogenetic tree construction

The representative sequences of highly abundant OTUs (relative abundance > 5% in one of the 10 samples) were aligned by MEGA 7 [25] (http://www.megasoftware.net/). Sixty-three sequences from Groups 8, 9, and 10 and from isolates and enrichment cultures were selected from the RIM-DB. The phylogenetic analysis was performed on the 762 bp-to-1099 bp region of the 16S rRNA genes via the Maximum Likelihood method based on the Kimura 2-parameter model. The tree was resampled 500 times. The dendrogram was rooted with four *Methanopyrus* sequences.

### Statistical analyses

The statistical calculations were carried out with appropriate tests using the SPSS software package

(SPSS v. 20.0, SPSS Inc., Chicago, IL, USA). The data are shown as means ± standard deviations. The goat was the experimental unit for all comparisons, and diet was regarded as the fixed effect. The normality of the distribution of variables was tested via the Shapiro–Wilk test. The independent samples *t*-test procedure was used to analyze the variables found to have a normal distribution. The variables found to have a non-normal distributions were analyzed using the Kruskal–Wallis test procedure. Significance was defined as *P* < 0.05.

## Results

The pH values of the rumen contents for the hay-diet animals (6.12 ± 0.09) were significantly higher than those for the high-grain-diet animals (5.33 ± 0.09, *P* < 0.05).

### The relative abundance of *Methanomassiliicoccales* in the goat rumen as measured via real-time PCR

The real-time PCR results showed that the relative abundance of *Methanomassiliicoccales* (vs. total archaea) in the rumen of goats fed the hay diet was 8.20% ± 2.43%, which was higher than that (0.50% ± 0.38%) in the rumen of goats fed the high-grain diet (*P* = 0.002).

### Diversity of *Methanomassiliicoccales* in goat rumen

**Fig. 1:**
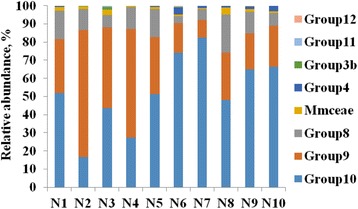
The relative abundance of *Methanomassiliicoccales* at the genus level based on the RIM-DB. N1 ~ 10, Name of each animal; Mmceae, Methanomassiliicoccaceae

Across all ten samples, 55,914 trimmed sequences were obtained. The average length of the trimmed sequences was 321 bp. Of these sequences, 51,345 were classified as *Methanomassiliicoccales*, and 180 were classified as Methanomicrobia or Methanobacteria or remained unclassified. Across all reads from the ten samples, a total of 208 OTUs were formed after the removal the OTUs containing less than ten sequences. All the sequences were classified as Methanomassiliicoccaceae at family level, and most of the sequences (96.82% ± 1.64%) were further classified as Group 8, 9, and 10 at the genus level of *Methanomassiliicoccales* based on the RIM-DB (Fig. 1).

Differences in diversity and evenness between the hay- and high-grain-diet groups were determined via an OTU-level-based calculation of the number of OTUs, as well as the Chao1, Shannon, and Pielou’s evenness indexes. No significant differences were noted between the two diet groups regarding for the number of OTUs (108.0 ± 19.9 vs. 86.4 ± 6.5, *P* = 0.050), Chao1 index (133.5 ± 22.9 vs. 115.9 ± 27.4, *P* = 0.302), Shannon index (3.98 ± 0.72 vs. 3.46 ± 0.19, *P* = 0.181), or Pielou’s evenness index (0.85 ± 0.12 vs. 0.77 ± 0.04, *P* = 0.261).

### The influences of diets on the *Methanomassiliicoccales* community

At the genus level (Fig. 1), the relative abundances of Group 10 (67.25 ± 12.76 vs. 38.13 ± 15.66, *P* = 0.012) and Group 4 (2.07 ± 1.30 vs. 0.27 ± 0.30, *P* = 0.035) were significantly higher in the high-grain-diet group, while the relative abundance of Group 9 was significantly higher in the hay-diet group (18.82 ± 6.20 vs. 47.14 ± 17.72, *P* = 0.020). There were no significant differences observed for Group 8 (10.00 ± 6.91 vs. 12.30 ± 3.65, *P* = 0.529) or unclassified Methanomassiliicoccaceae (1.51 ± 1.24 vs. 1.57 ± 0.86, *P* = 0.0.934) between the two diet groups.

**Fig. 2:**
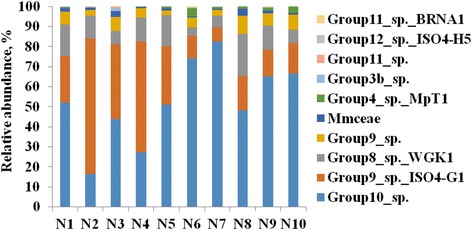
The relative abundance of *Methanomassiliicoccales* at the species level based on the RIM-DB. N1 ~ 10, Name of each animal; Mmceae, Methanomassiliicoccaceae

At the species level (Fig. 2), the relative abundance of Group 10 sp. (67.25 ± 12.76 vs. 38.13 ± 15.66, *P* = 0.012) and Group 4 sp. MpT1 (2.07 ± 1.30 vs. 0.27 ± 0.30, *P* = 0.035) were significantly higher in the high-grain diet group, while the relative abundance of Group 9 sp. ISO4-G1 was significantly higher in the hay-diet group (12.83 ± 3.87 vs. 42.44 ± 18.47, *P* = 0.022). There was no significant difference observed for Group 8 sp. WGK1 (10.00 ± 6.91 vs. 12.30 ± 3.65, *P* = 0.529), Group 9 sp. (5.99 ± 2.34 vs. 4.70 ± 2.10, *P* = 0.387) and unclassified Methanomassiliicoccaceae (1.51 ± 1.24 vs. 1.57 ± 0.86, *P* = 0.0.934) between the two diet groups.

**Fig. 3:**
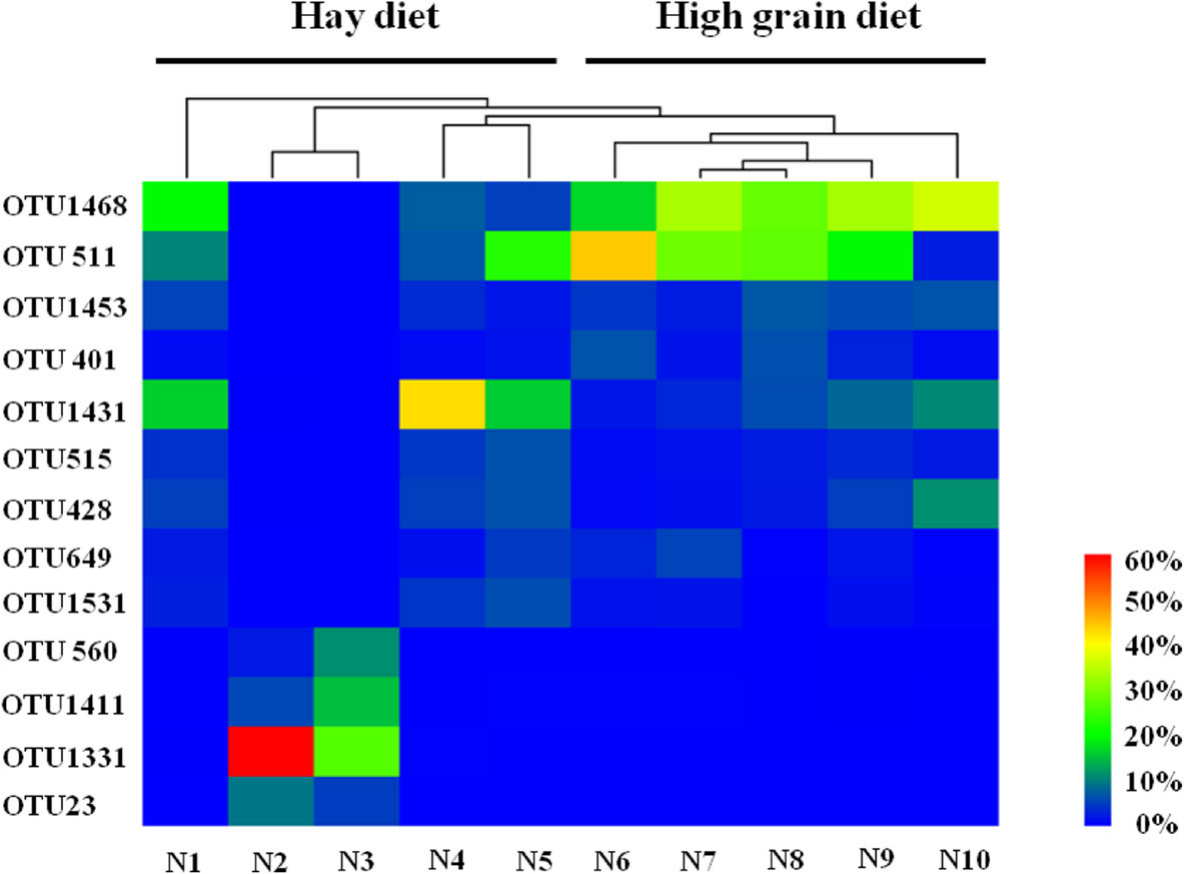
Hierarchical clustering of samples based on the relative abundance of each OTU in each sample. Only OTUs with a relative abundance of at least 5% in one of the ten samples were included. The names of animals are shown below the heat map (N1 ~ 10). Heat map colors represent the relative abundance of OTUs (%)

At the OTU level (Fig. 3), among the dominant OTUs (≥5% of the relative abundance in at least one of the samples), only the relative abundances of OTU1468 (belonging to Group 10, 30.28% ± 7.93% vs. 6.61% ± 8.29%, *P* = 0.002) was significantly higher in the high-grain-diet group.

**Fig. 4:**
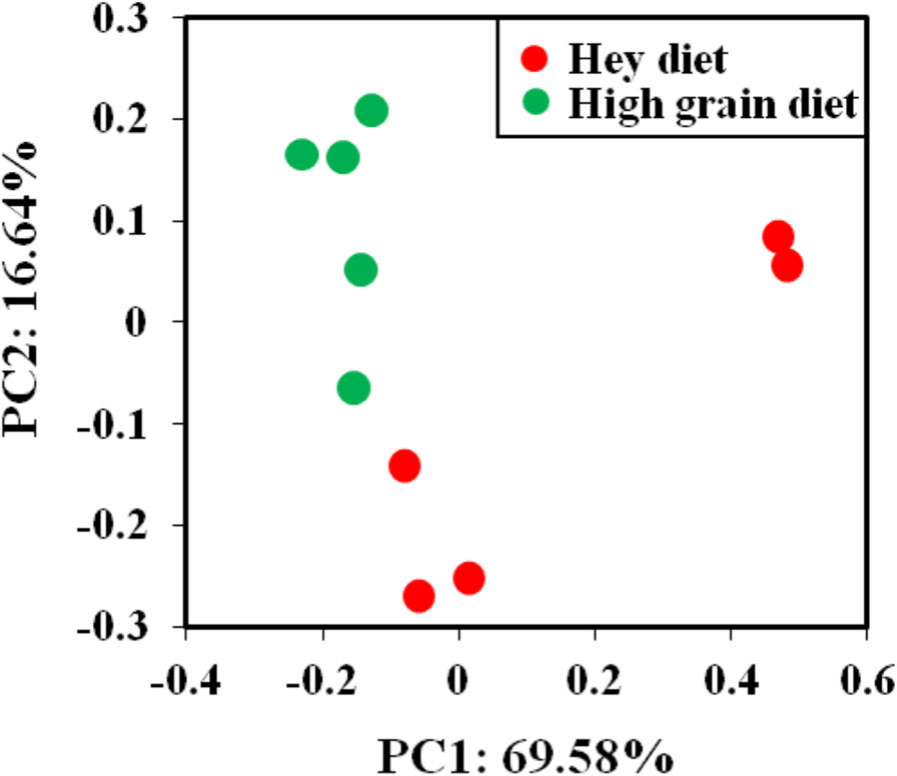
Effects of diet on *Methanomassiliicoccales* community structure. A principal coordinate analysis (PcoA) plot of unweighted unifrac dissimilarities is shown

Principal coordinate analysis (PCoA) plots based on unweighted UniFrac distance metrics showed that the community of *Methanomassiliicoccales* was altered by the hay- and high-grain-diet using PC1 and PC2 (69.58% and 16.64%, respectively, of the explained variance, Fig. 4). The hay-diet group displayed relatively larger individual differences, as compared with the high-grain diet group.

### Identification and phylogenetic analysis of dominant OTUs in *Methanomassiliicoccales*

**Fig. 5:**
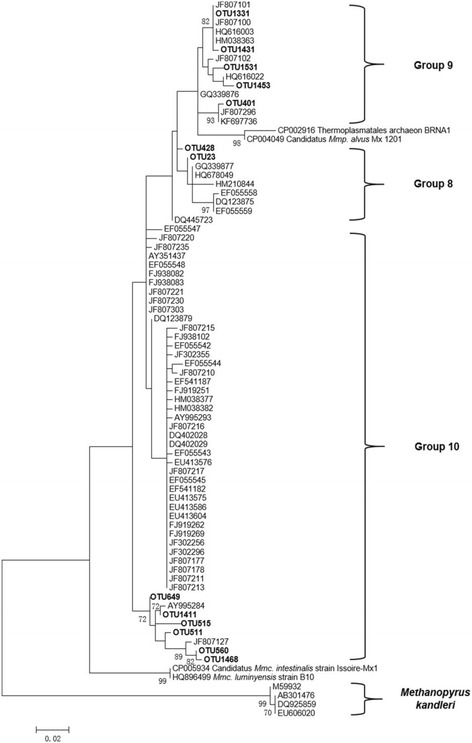
Phylogeny of *Methanomassiliicoccales* sequences and highly abundant OTUs. Representative sequences of highly abundant OTUs (bold, ≥5% of the relative abundance in one of the ten samples) were aligned by MEGA 7. The GenBank accession numbers of these highly abundant OTUs are KX525649–61. Sixty-three sequences from Groups 8, 9, and 10 and isolates and enrichment cultures were selected in the RIM-DB. Phylogenetic analysis was performed on the 762 bp-to-1099 bp region of the 16S rRNA genes via the Maximum Likelihood method based on the Kimura 2-parameter model. The tree was resampled 500 times, and only bootstrap values of ≥70% are shown. The dendrogram was rooted with four *Methanopyrus* sequences. The scale bar indicates 0.02 inferred nucleotide substitutions per position. *Mmc*., *Methanomassiliicoccus*; *Mmp*., *Methanomethylophilus*

Thirteen predominant OTUs contributed 71.41% (±8.15%) of the total relative abundance of *Methanomassiliicoccales* in the hay-diet group and 79.79% (±3.84%) in the high-grain-diet group. A phylogenetic analysis of the representative sequences from the 13 predominant OTUs revealed that all 13 OTUs were situated in Groups 8, 9, and 10 (Fig. 5). The representative sequences of the two most highly abundant OTUs in each group were blasted in GenBank. The most closely related sequences of these OTUs were identified, and all were uncultivated representatives. OTU1331 and OTU1431 had the highest mean relative abundance (17.84% ± 27.11% and 15.43% ± 17.87%) in the hay-diet group and were situated in *Methanomassiliicoccales* Group 9. OTU1331 and OTU1431 were 98% and 97% similar to sheep rumen mixed culture ISO4-G1 (CP013703), respectively. OTU1331 was widely distributed among ruminants; it was 100% similar to water buffalo rumen clone BBC-21 (JF9517736), *Budorcas* rumen clone f142b (KM650116), sika deer rumen clone SDmet98 (KC454162), cattle rumen clone QTPC108 (JF807170), yak rumen clone QTPYAK64 (JF807240), and other clones. OTU1431 showed 99% similarity to the above clone sequences. OTU511 and OTU1468 had the highest relative abundance (25.07% ± 15.63% and 30.28% ± 7.93%) in the high-grain-diet groups and were situated in Group10. OTU511 was 96% similar to *Candidatus Methanoplasma termitum* strain MpT1 and 99% similar to the well-known sheep rumen clone NZCRCC002 (HM624057), feedlot cattle clone ON-CAN.16 (DQ123886), and other clones. No cultured representatives are currently available for Group 10. OTU1468 was 94% similar to *Ca. M. termitum* strain MpT1 and 99% similar to water buffalo rumen clone G-56 (AB906274), sika deer rumen clone SDcsmet90 (KC454244), and other clones.

## Discussion

A comprehensive understanding of the archaeal communities in the rumen is important for rumen methane mitigation and the improvement of animal performance. For the *Methanomassiliicoccales,* as a new archaeal order, information on community and taxonomy remains limited. St-Pierre and Wright [5] reported substantial variability in the abundance of *Methanomassiliicoccales* in the rumens of different animals. The present study used real-time PCR, as well as a pyrosequencing approach and the RIM-DB database, to describe the abundance and community of *Methanomassiliicoccales* in the rumens of Chinese goats. These rumens had a relatively low abundance of *Methanomassiliicoccales* in the archaeal community, which complicated the sequencing of the 16S rRNA. For this reason, nested PCR was employed in the present study for the amplification of the 16S rRNA gene sequences of *Methanomassiliicoccales* in the goat rumen contents. However, it should be noted that performing nested PCR prior to in-depth sequencing may introduce biases regarding estimated microbial diversity and community structure, especially for communities with relatively higher levels of diversity [26]. Compared with standard PCR, nested PCR may yeild fewer OTUs, lower Shannon and Chao1 indeces, as well as increases or decreases in the relative abundances of some taxa [26].

Pyrosequencing targeted at the 16S rRNA genes of this new methanogenic order revealed a diverse community of *Methanomassiliicoccales* in the goat rumen, which was consistent with previous findings for sheep, cattle, and red deer in New Zealand obtained using the denaturing gradient gel electrophresis (DGGE) method [14]. To date, only one pure strain and a few enriched cultures have been described in this order, and no type strains are available for most of the phylogenetic groups of *Methanomassiliicoccales*. This limited further taxonomic analysis of the sequences in the present study. Most sequences were assigned to one of three groups (Groups 8, 9, and 10) based on the RIM-DB [12], which was inconsistent with the findings of Seedorf et al. [6, 12], in which most of the ruminal sequences from New Zealand sheep and cattle were assigned to Groups 10, 11, and 12. This difference should be taken into account when the methane mitigation tools, such as vaccines or small-molecule inhibitors, are developed for various ruminants.

The most highly abundant OTUs in the goat rumen were uncultured. The two most highly abundant OTUs in the hay-diet group were different from the two most highly abundant OTUs in the high-grain-diet group. A high-grain diet is a well-known general cause of pH decreases in the rumen, which could suppress methanogens that are sensitive to low pH values [27]. Thus, the four species may have specific properties that allowed their survival in their corresponding habitats. The genome of *Ca. M. alvus* encodes a choloylglycine hydrolase that imparts resistance to bile salts encountered in the GIT [28]. Another adaptation to GIT could be inferred by the presence of a conserved amino acid domain corresponding to COG0790. *Ca. M. alvus* encoded 28 genes related to the proteins within this domain, *Ca. M. intestinalis* encoded six such genes, and *M. luminyensis* encoded one, which suggests differences in the adaptation of these three species to the digestive tract [28]. Söllinger et al. [10] reported larger genomes and a larger number of genes encoding anti-oxidative enzymes in the members of *Methanomassiliicoccales* from the ‘environmental’ clade than in those from the GIT clade, indicating that the GIT members were restricted and specialized to the GIT habitat. Further isolation and genomic analysis may help to reveal the physiological properties of these novel methanogens, as well as their genomic adaptation to their habitats.

The high-grain diet decreased the relative abundance of *Methanomassiliicoccales* in the rumen, possibly due to its pH-lowering effect in the rumen, suggesting that the growth of *Methanomassiliicoccales* was depressed. Methylamines and methanol are the substrates of *Methanomassiliicoccales* [1, 8, 9]. The suppression of the growth of *Methanomassiliicoccales* in the high-grain-diet group might cause an accumulation of these methyl compounds in the rumen. Ametaj et al. [29] observed an accumulation of methylamines and methanol in the rumen liquid with the increasing proportions of cereal grain in the diet. It is worthwhile to note the effect of the accumulation of these methyl compounds on the physiology of the rumen and the health of the host animals.

The relative abundances of *Methanomassiliicoccales* in the rumen contents of Chinese goats were 0.5 ± 0.2% with the high-grain diet and 8.2 ± 1.1% with the hay diet. St-Pierre and Wright [5] described a wide variation (~80.8%) in the relative abundance of ruminal *Methanomassiliicoccales* based on host species, diet, and geographical locations. Seedorf et al. [6] reported that *Methanosphaera* and *Methanomassiliicoccales* OTUs were highly abundant in lucerne-fed sheep as compared to pasture-fed sheep. This high abundance may be related to the levels of the precursors of the substrates in the animal’s diets, but this requires further confirmation. The exact factors contributing to the high proportion of *Methanomassiliicoccales* in some animals remains unclear.

The communities of *Methanomassiliicoccales* were altered at OTU level by diet using PcoA analysis and hierarchical clustering, respectively. Apart from the low pH, the observed differences may be related to changes in the community structure of the other prominent microbial organism groups (bacteria, protozoa, and fungi). Previous studies have reported a close relationship between *Methanomassiliicoccales* species and protozoa [30] and fungi [31]. Most of the cultured *Methanomassiliicoccales* species were enriched with bacteria that were difficult to remove, which suggested a close interaction between them. Information is limited regarding the relationships between *Methanomassiliicoccales* species and other rumen microorganisms, but this information is important in the effect to reveal the factors affecting the *Methanomassiliicoccales* community. A co-culture approach may be a feasible way of studing these relationships [31].

## Conclusion

The data presented here have shown that at the genus level, only a few groups were dominant in the Chinese goat rumen. Overall, *Methanomassiliicoccales* were present at a low relative abundance among the methanogen community; therefore, they are probably less important contributors to the rumen methane emission of the Chinese goats. Furthermore, the abundance of *Methanomassiliicoccales* in the rumen was easily disturbed by dietary changes. A better understanding of the community of *Methanomassiliicoccales* in the rumen could help to identify the role of this new order in the rumen.
